# Pesticides: In Search of a Better Mosquito Repellent

**DOI:** 10.1289/ehp.116-a337

**Published:** 2008-08

**Authors:** Carol Potera

Insect bites are more than just an itchy nuisance; mosquitoes and ticks spread malaria, West Nile virus, Lyme disease, and other illnesses. The insect repellent DEET keeps insects away, but it wears off with sweat and can cause health problems in sensitive people, including rashes, skin and mucous membrane irritation, dizziness, headaches, disorientation, and nausea. Although DEET shows broad-spectrum activity against biting insects, it apparently does not work as well against malaria-carrying strains of mosquitoes. Finally, DEET smells bad, and it can damage plastic eyeglass lenses and watch faces. Now scientists are studying a new type of repellent that overcomes at least one of these myriad disadvantages, lasting three times longer than DEET.

For the past 60 years, the U.S. Department of Agriculture (USDA) has tested tens of thousands of chemicals as possible repellents and catalogued the results. Recently, researchers at the USDA Agricultural Research Service and the University of Florida evaluated the records of several hundred diverse compounds and focused on the most effective ones, all of which were acylpiperidines—chemical cousins of the compound piperidine, which gives black pepper its bite. They selected 11 compounds with confirmed effectiveness, and chemists in the group synthesized 23 more, guide by a computer model that predicts improved performance by altering chemical groups.

The mosquito-repelling potency of each compound was tested by volunteers who put their arms inside boxes filled with *Aedes aegypti* mosquitoes for 1 minute per compound per day, while the researchers recorded how many days it took to be bitten. Under these test conditions, the DEET treatment wore off after 17.5 days on average, whereas several of the test compounds lasted 40 to 50 days, and one remained active for 73 days. The results, reported in the 27 May 2008 issue of the *Proceedings of the National Academy of Sciences*, “were just phenomenal,” says Ulrich Bernier, the USDA research chemist who conducted the bite tests.

Next, Bernier and colleagues will evaluate the effectiveness of the compounds against mosquitoes that transmit malaria, and then against other insect species. The best repellents will then be assessed for skin toxicity. “We hope the acylpiperidines are safer than DEET, but we don’t know yet,” says Bernier.

The same USDA chemical collection that contained the acylpiperidines also spawned DEET, which has been the reigning repellent since its commercialization in the 1950s. DEET is a very good repellent, says Bernier, despite its drawbacks. To be economically viable, any competitor not only must repel insects better than DEET, but also overcome its disadvantages. So why haven’t the newly identified compounds been pursued further in the past? Bernier explains that, efficacy-wise, “they were equal to or just a bit better than DEET. . . . It would not be advisable to spend millions of dollars just to introduce a repellent into the market that one advertises as ‘about as good as DEET.’”

“There’s always room for new and effective repellents, because consumers want a choice,” says Jonathan Day, a medical entomologist at the University of Florida in Vero Beach. Even during medical emergencies such as an outbreak of West Nile virus, some people refuse to use DEET, according to Day, and newer botanical formulations do not repel insects as well. The acylpiperidines look promising, but it’s a long road from identifying a potential repellent to registration with the Environmental Protection Agency. “Time will tell whether these compounds make it to store shelves,” Day says.

